# Supplementary figures and images for: An oxytocin/vasopressin-related neuropeptide modulates social foraging behavior in the clonal raider ant
Source: PLoS Biol. 2021 Jun 30;19(6):e3001305. doi: 10.1371/journal.pbio.3001305 (PMC8244912; doi:10.1371/journal.pbio.3001305)

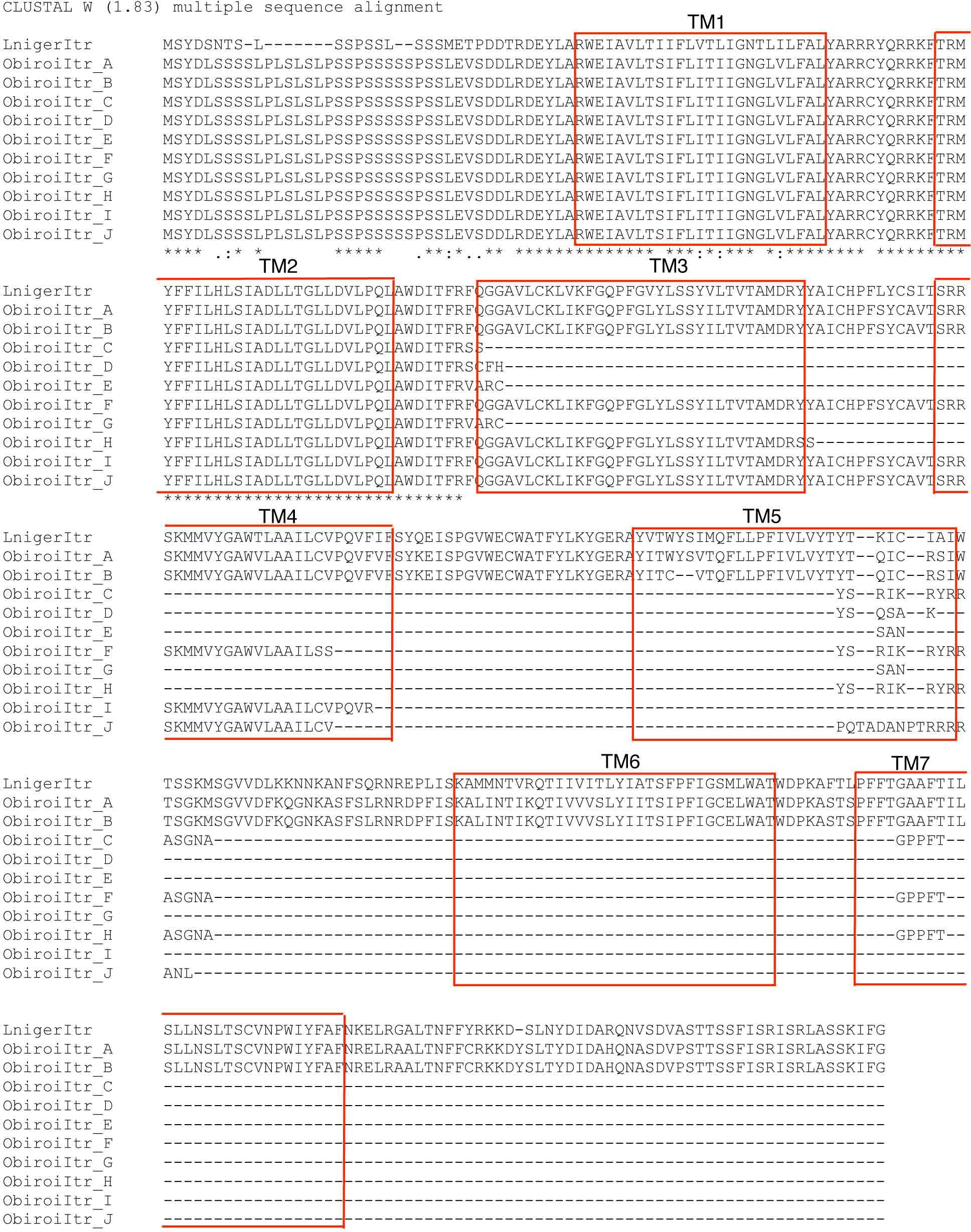

Supplement: S1 Fig — Alignment of the inotocin receptor splice variants inferred from RNA-seq data [52], along with the Lasius niger inotocin receptor as a reference (from [56]). The transmembrane domains required for proper receptor function are highlighted in red. The data underlying this figure can be found in S4 Data. RNA-seq, RNA sequencing. (TIF) [file pbio.3001305.s001.tif]

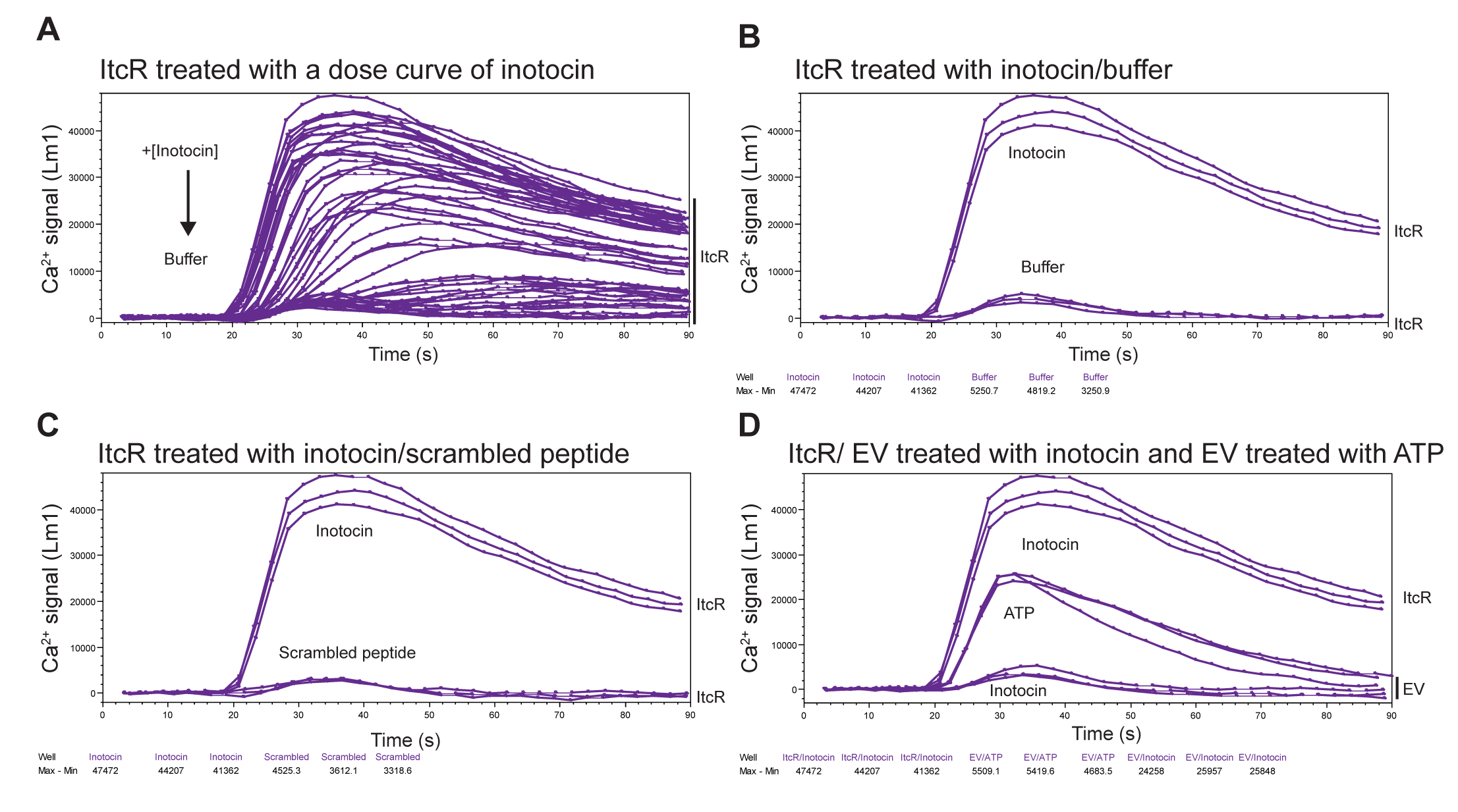

Supplement: S2 Fig — (A) The ItcR responds in a dose-dependent manner to inotocin. (B) No response is detected when stimulated with the vehicle alone (buffer). (C) No Ca2+ influx is detected when ItcR expressing cells are stimulated with a scrambled control peptide. (D) No Ca2+ influx is detected when cells transfected with an empty vector are stimulated with inotocin (100 nM), and cells transfected with an empty vector are healthy and show calcium responses when stimulated with ATP (10 μM). The traces for the inotocin response when stimulating the inotocin receptor are the same data displayed in each panel. The data underlying this figure can be found in S5 Data. HEK293T, human embryonic kidney 293T. (TIF) [file pbio.3001305.s002.tif]

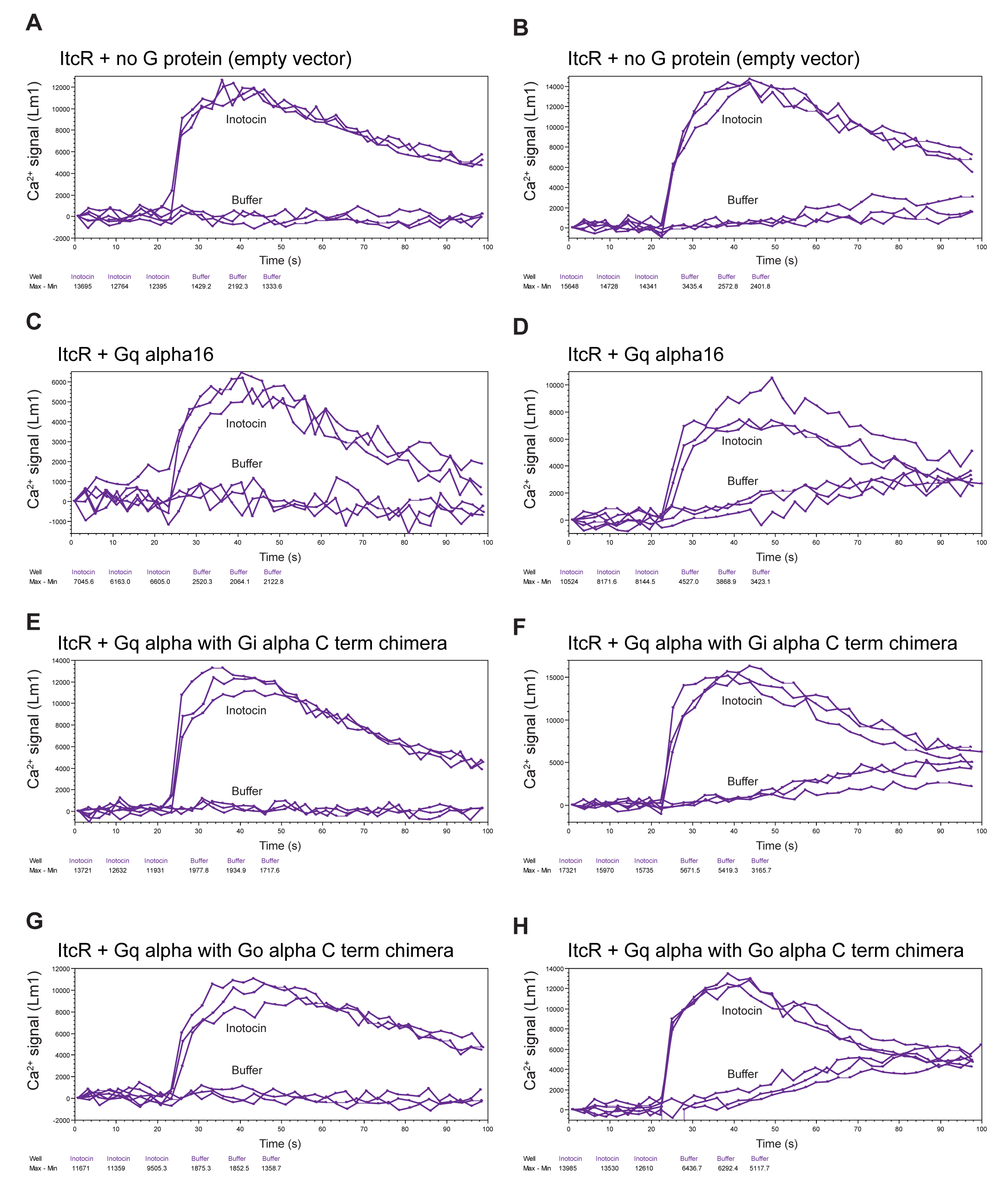

Supplement: S3 Fig — (A and B) itcR co-transfected with empty vector (no G protein) show calcium responses when stimulated with inotocin. (C and D) Co-transfection with Gq alpha 16. (E and F) Co-transfection with Gq alpha with Gi alpha C term chimera. (G and H) itcR co-transfection with Gq alpha with Go alpha C term chimera. In A, C, E, and G cells were grown at 37°C for 24 hours and then transferred to 28°C for an additional 16 to 24 hours. In B, D, F, and H cells where constantly grown at 37°C for the same amount of total time. The data underlying this figure can be found in S6 Data. HEK293T, human embryonic kidney 293T. (TIF) [file pbio.3001305.s003.tif]

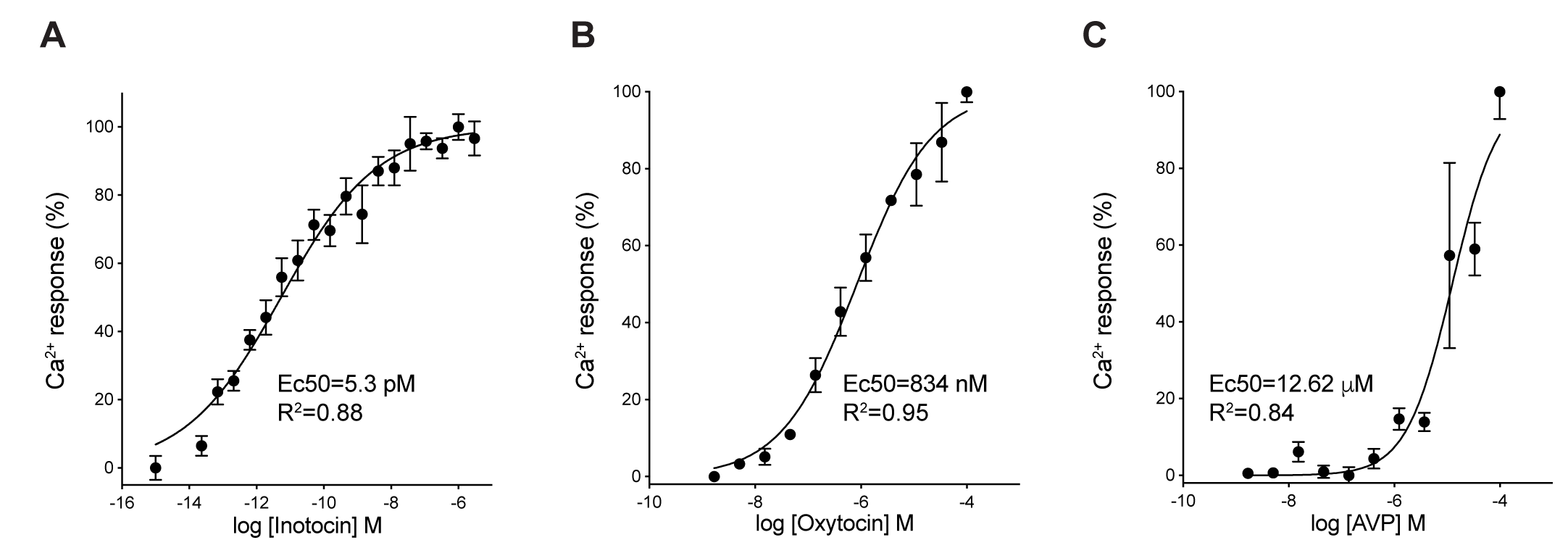

Supplement: S4 Fig — (A) Inotocin Ca2+ response in cells transfected with itcR (independent experiment from the one displayed in Fig 1C). (B) Oxytocin Ca2+ response in cells transfected with itcR. (C) AVP Ca2+ response in cells transfected with itcR. Experiment shown in A is one of 3 replicates, and each concentration of inotocin was assayed in sextuplicate in all replicates. Experiments in B and C were performed once and include triplicate measurments. Previous experiments have shown that HEK293T cells do not endogenously express the oxytocin or vasopressin receptors (Supporting information references [1–5] in S1 Text). The data underlying this figure can be found in S7 Data. See S1 Text for Supporting information methods. AVP, arginine vasopressin; HEK293T, human embryonic kidney 293T. (TIF) [file pbio.3001305.s004.tif]

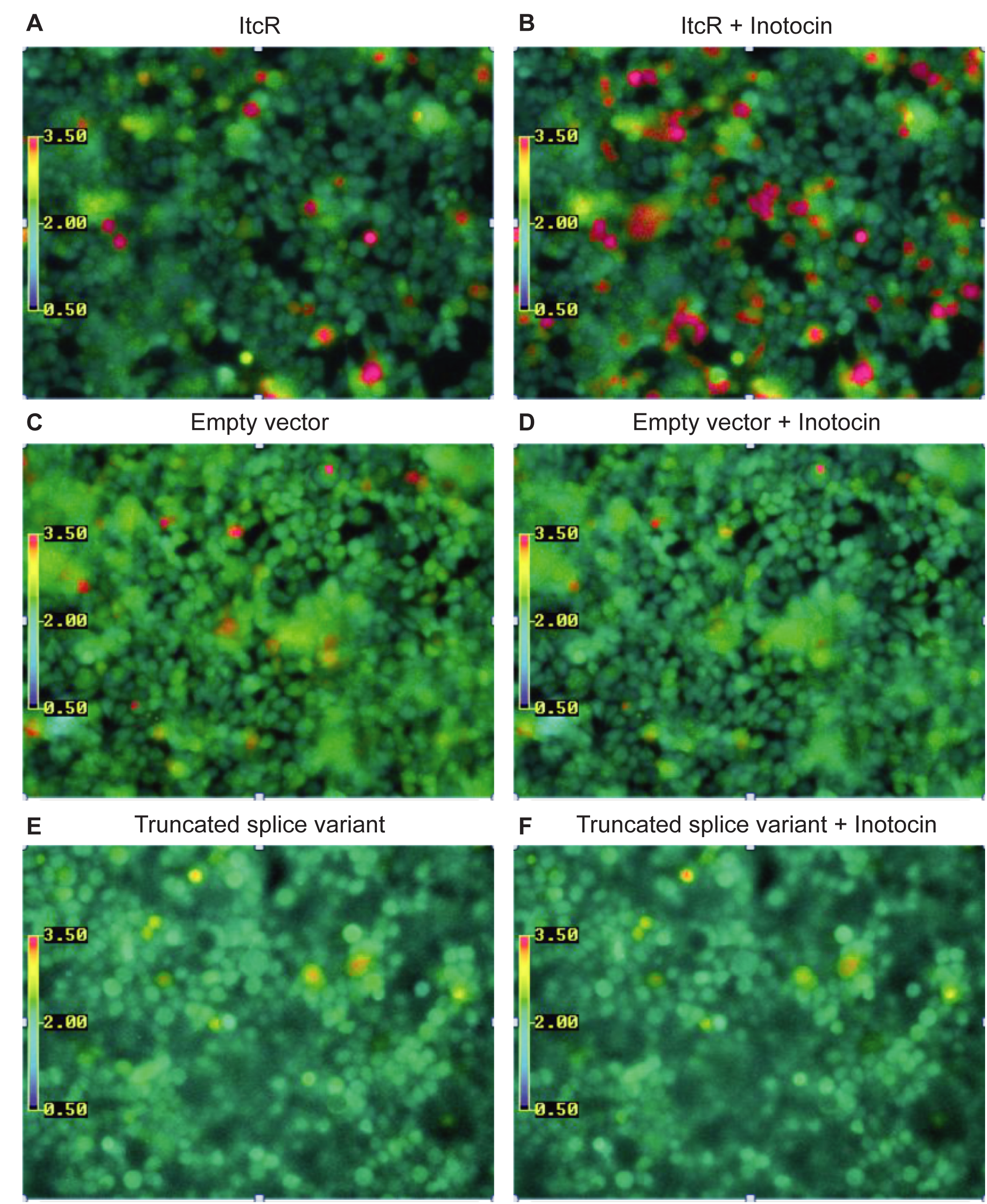

Supplement: S5 Fig — Lack of response in cells transfected with an empty vector or with a truncated receptor isoform. Each image is one frame extracted from recordings of HEK293T cells stimulated with inotocin. The panel on the left is from before stimulation, and the panel on the right is from after the introduction of inotocin. (A and B) Calcium signal is detected (red) in cells expressing the inotocin receptor (ObiroiItr_A, for protein sequence see S1 Fig) and G protein 16. (C and D) No calcium signal is detected when stimulating the cells transfected with an empty vector. (E and F) No calcium signal is detected when a truncated splice variant of the receptor is expressed (ObiroiItr_H, which lacks TM4-7, for protein sequence see S1 Fig). See S1 Text for Supporting information methods. HEK293T, human embryonic kidney 293T. (TIF) [file pbio.3001305.s005.tif]

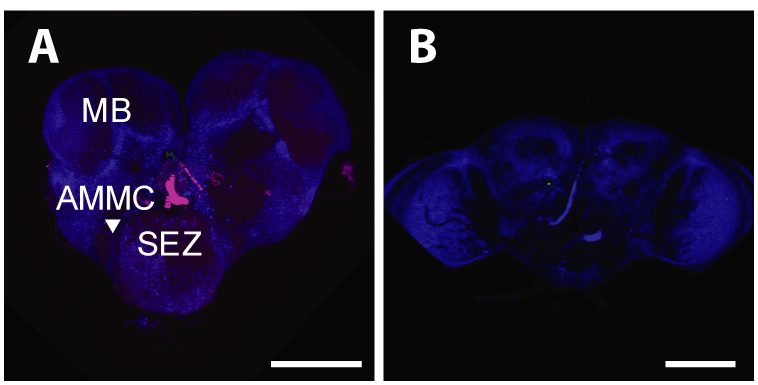

Supplement: S6 Fig — (A) Control staining without primary antibody. (B) A Drosophila melanogaster brain stained with the inotocin antibody (green). Drosophila melanogaster lacks the inotocin system, and, accordingly, no antibody staining is visible. Nuclei are stained with DAPI (blue), and actin is stained with phalloidin (magenta). Scale bar in each panel represents 100 μm. AMMC, antennal mechanosensory and motor center; MB, mushroom body; SEZ, subesophageal zone. (TIF) [file pbio.3001305.s006.tif]

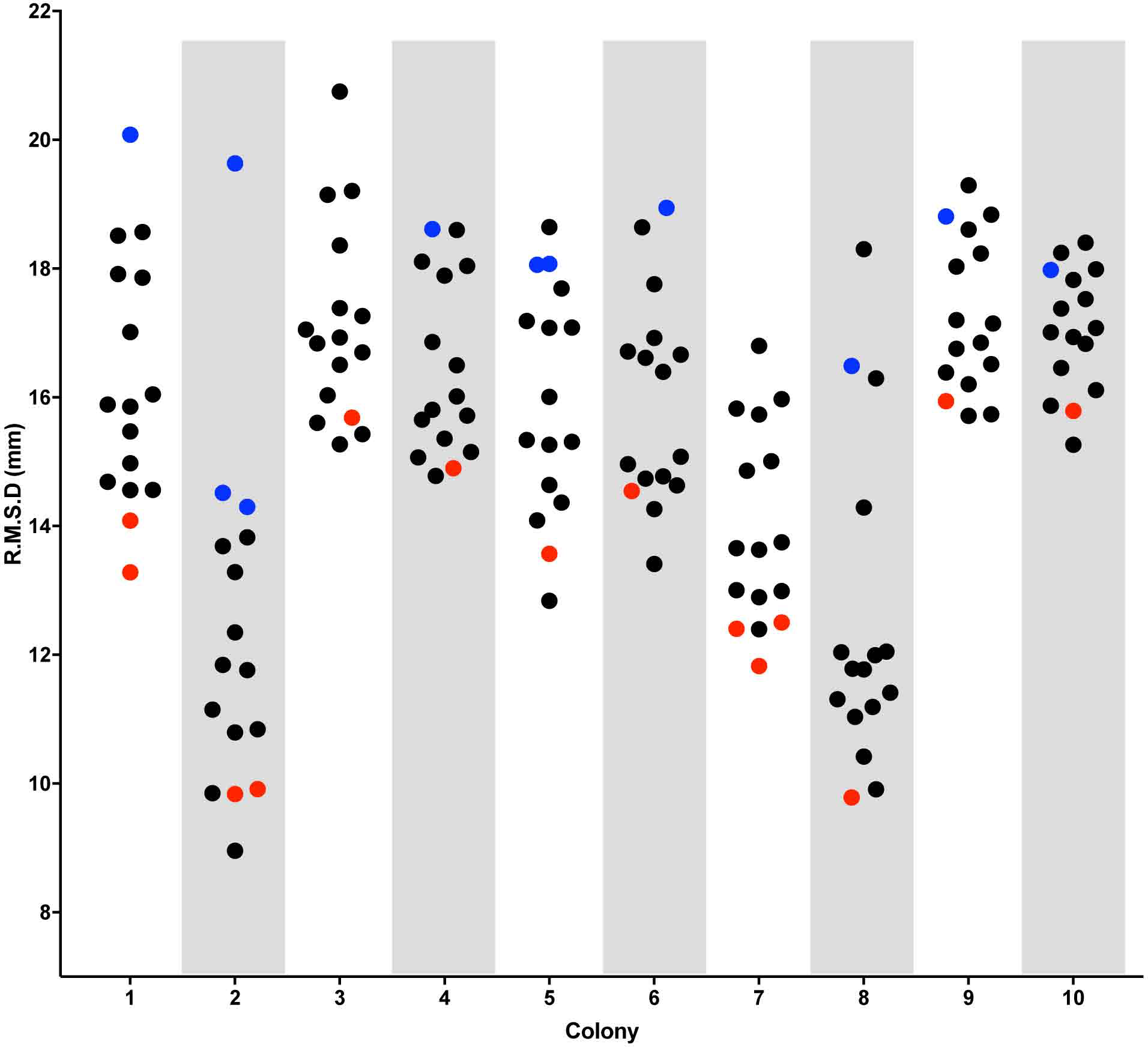

Supplement: S7 Fig — Ten colonies of 16 ants each were monitored using automated behavioral tracking, and ants were ranked according to their r.m.s.d. values (see Materials and methods). Individuals used for inotocin measurements (Fig 3A) are shown in red (nurses) and blue (foragers). We were not able to always include the most extreme workers and the same number of workers from all colonies, because some brains were lost or damaged during dissection, staining, or imaging. The data underlying this figure can be found in S8 Data. r.m.s.d., root–mean–square deviation. (TIF) [file pbio.3001305.s007.tif]

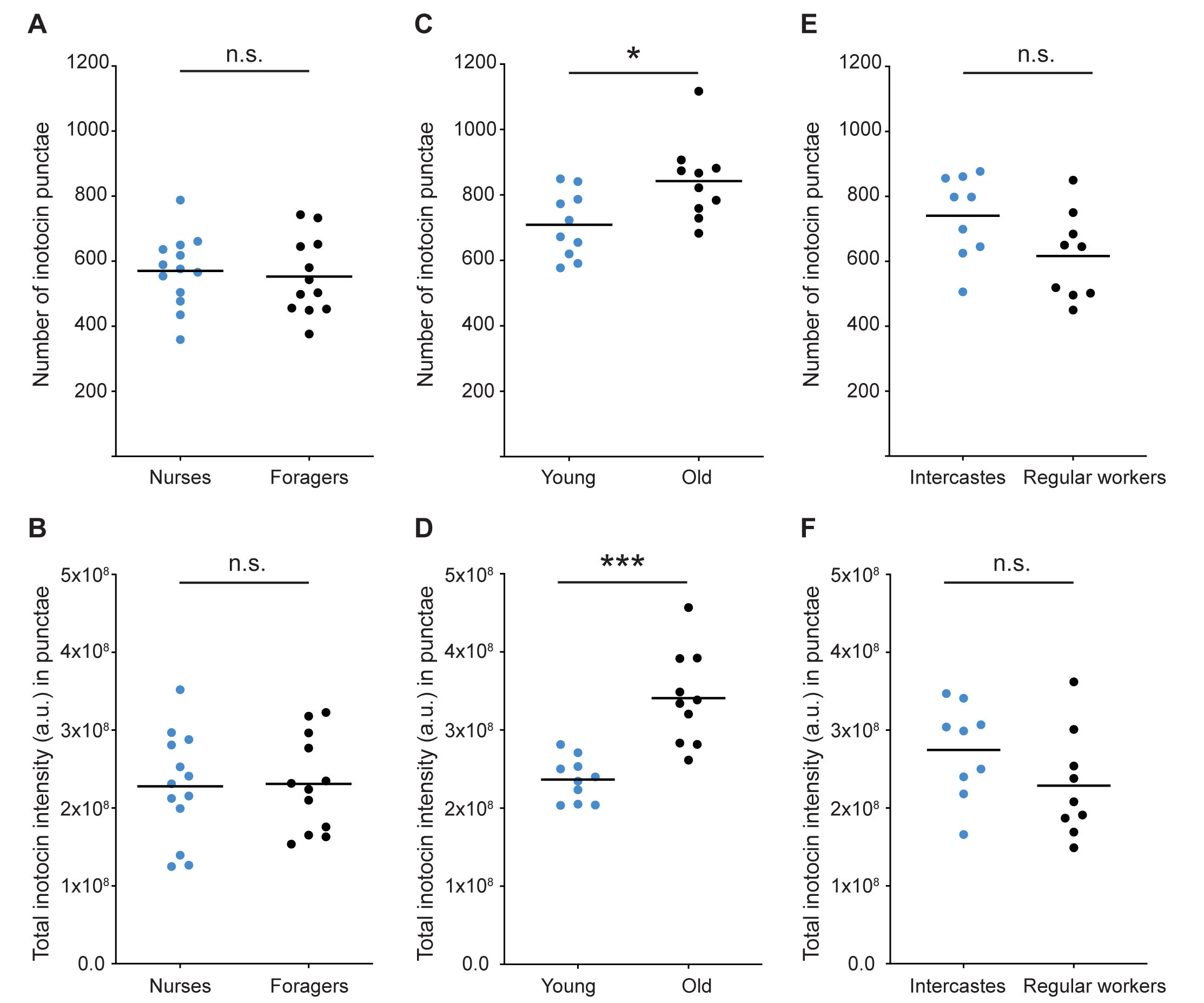

Supplement: S8 Fig — (A and B) No differences were found between age-matched nurses and foragers (n ≥ 12; unpaired t test, Bonferroni corrected). (C) Older ants have more inotocin punctae than young ants (n ≥ 10; unpaired t test, *p = 0.045, Bonferroni corrected). (D) Old ants show more inotocin staining (a proxy for the amount of inotocin) than young ants (n = 10; unpaired t test, ***p < 0.0003, Bonferroni corrected). (E and F) No differences were found between age-matched intercastes and regular workers (n ≥ 9; unpaired t test, Bonferroni corrected). The data underlying this figure can be found in S2 Data. (TIF) [file pbio.3001305.s008.tif]

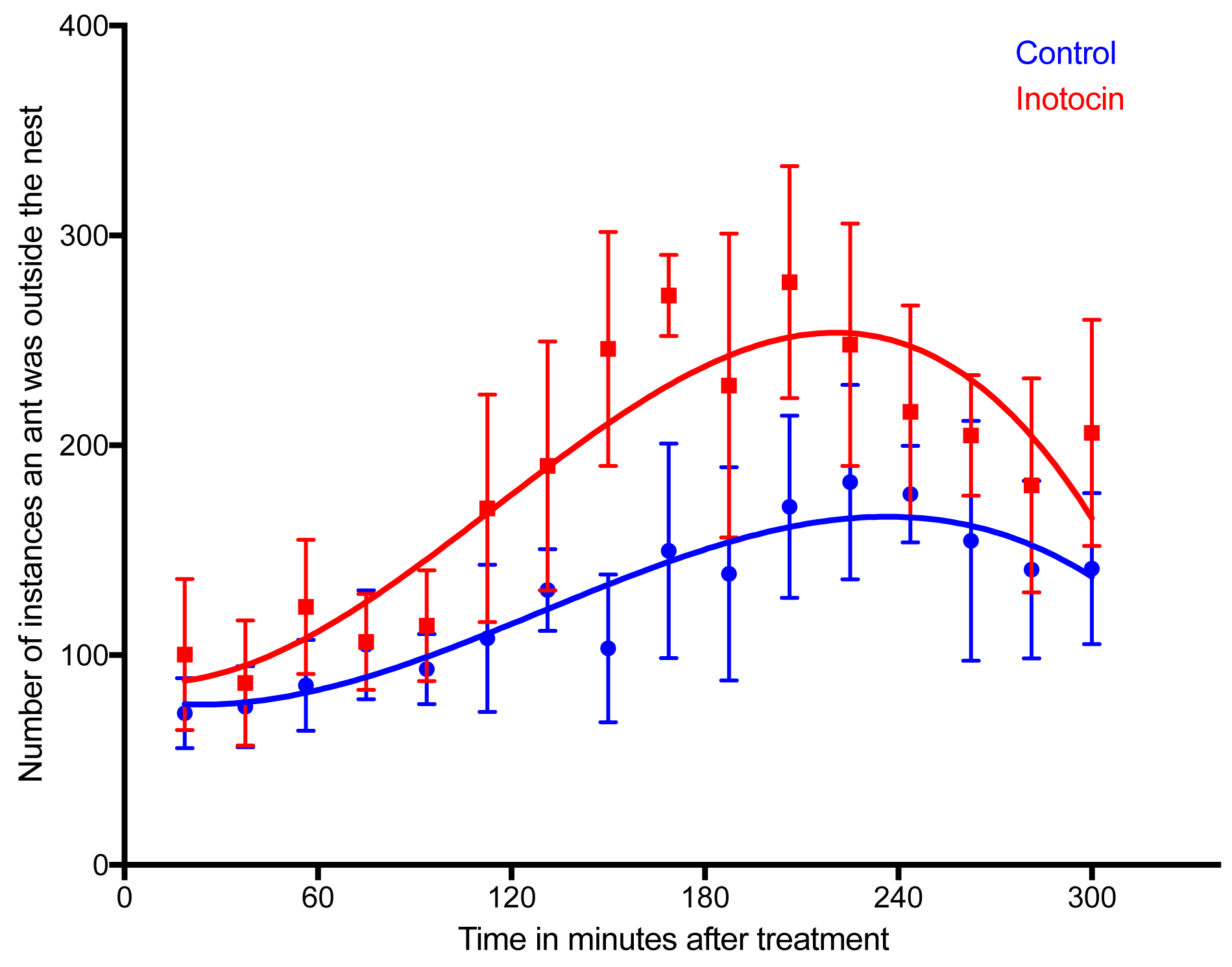

Supplement: S9 Fig — Total number of instances that ants treated with inotocin (red) or a control peptide (blue) were found outside the nest (a proxy for foraging activity) in a projection image of 500 frames corresponding to 18.75-minute windows over the course of 5 hours. Error bars show the standard error. In colonies composed of adult ants of varying ages with larvae, ants foraged more when treated with inotocin than when treated with the control peptide. An average of all ants per treatment per time point was graphed, and a second degree polynomial curve was fitted via nonlinear regression using PRISM GraphPad. The fitted curves were compared using the extra sum-of-squares F-test (n = 6; p = 0.0002). The data underlying this figure can be found in S9 Data. (TIF) [file pbio.3001305.s009.tif]

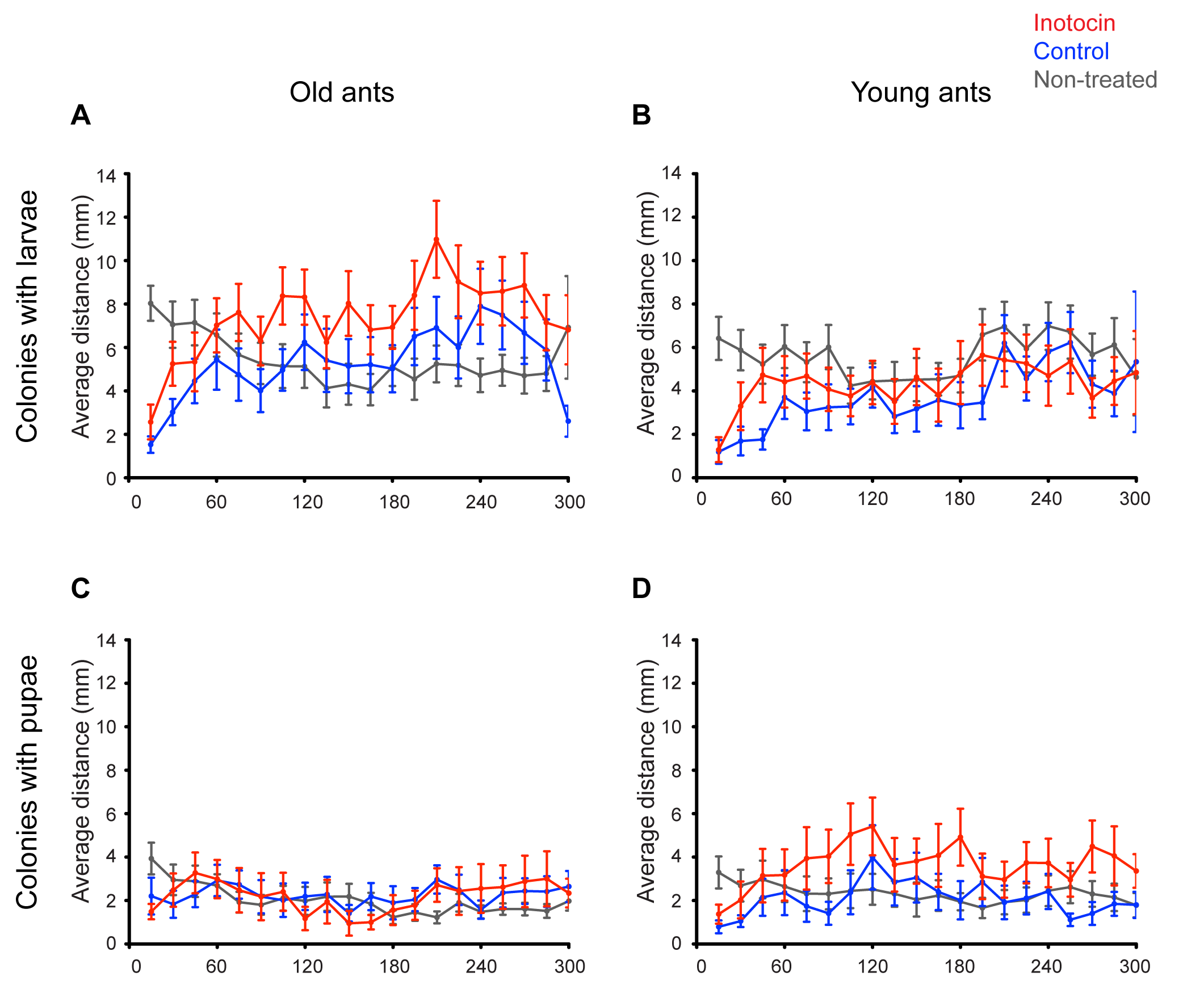

Supplement: S10 Fig — The data here are also depicted in Fig 4, with the inclusion here of the results for untreated ants (gray) that were part of the colony. Average distance traveled by ants in 15-minute time windows over the course of 5 hours (proxy for foraging activity) following pharmacological treatment (n = 20 ants for each treatment from 10 replicate colonies with larvae and 10 colonies with pupae; error bars show the standard error). (A and B) In the presence of larvae, old ants treated with inotocin (red) had higher average distance traveled than ants under any other experimental condition. (C and D) In colonies with pupae, only young ants treated with inotocin (red) increased their average distance traveled compared to ants under any other experimental condition. Control treated (blue) and non-treated (gray). Non-treated ants show relatively higher activity early in the experiment because experimental ants are still recovering from the immersion treatment. The data underlying this figure can be found in S3 Data and https://doi.org/10.5281/zenodo.4813078. (TIF) [file pbio.3001305.s010.tif]

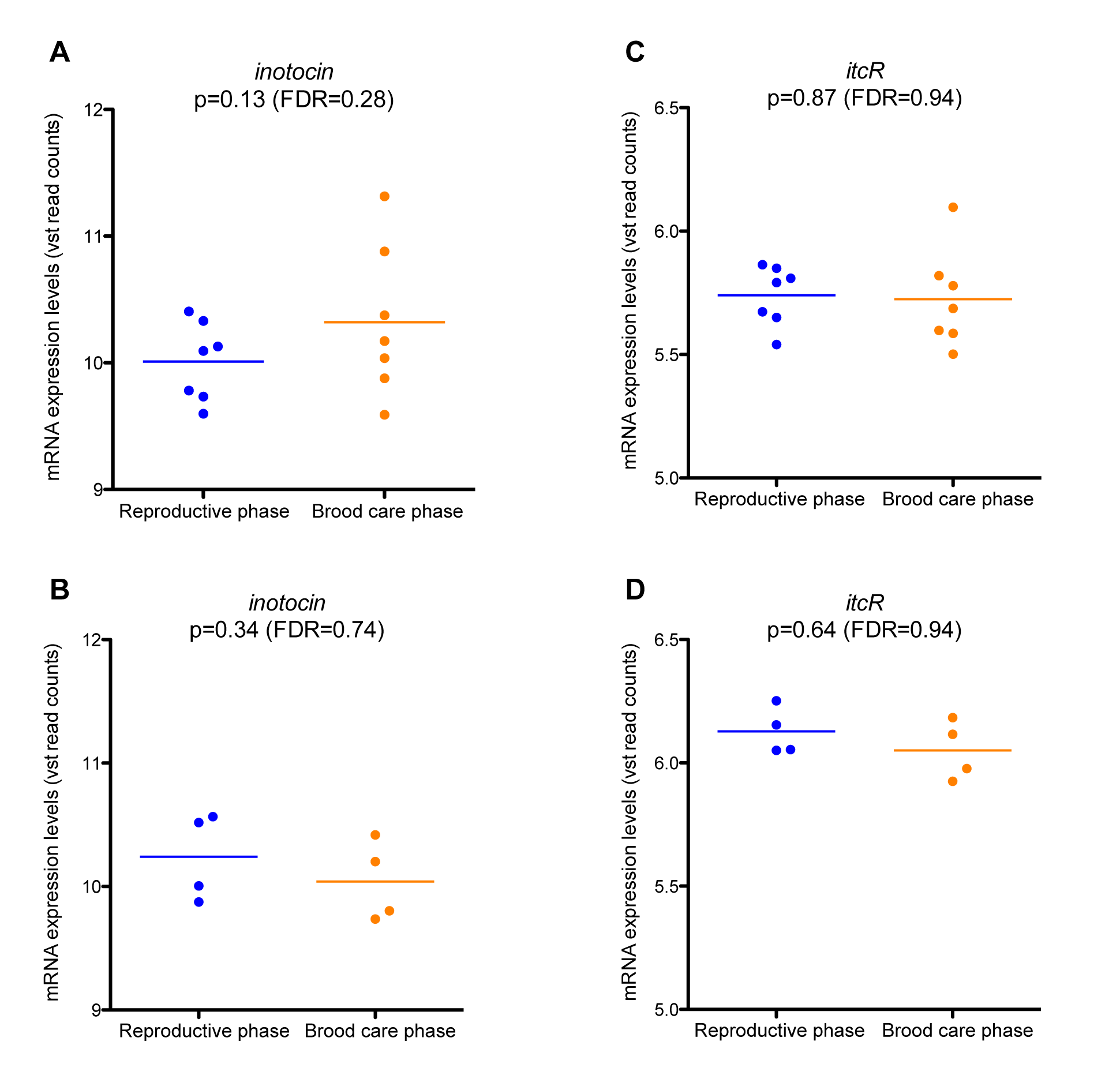

Supplement: S11 Fig — Gene expression data for inotocin (A and B) and itcR (C and D) were analyzed from the control samples originally used in [52,73]. Data from [52] for A and C and data from [73] for B and D. Samples in each case consist of age-matched workers in the brood care phase with larvae or age-matched workers in the reproductive phase with pupae. Wald tests were performed on each group to calculate significance. Gene expression units represent variance-stabilized transformed read counts. The data underlying this figure can be found in S10 Data and in https://doi.org/10.5281/zenodo.4562942. See S1 Text for Supporting information methods. (TIF) [file pbio.3001305.s011.tif]
