# Supplementary material for: An oxytocin/vasopressin-related neuropeptide modulates social foraging behavior in the clonal raider ant
Source: PLoS Biol. 2021 Jun 30;19(6):e3001305. doi: 10.1371/journal.pbio.3001305 (PMC8244912; doi:10.1371/journal.pbio.3001305)
Supplement: S4 Data — (RTF) [file pbio.3001305.s015.rtf]

CLUSTAL W (1.83) multiple sequence alignmentLnigerItr       MSYDSNTS-L-------SSPSSL--SSSMETPDDTRDEYLARWEIAVLTIIFLVTLIGNTLILFALYARRRYQRRKFTRMObiroiItr_A     MSYDLSSSSLPLSLSLPSSPSSSSSPSSLEVSDDLRDEYLARWEIAVLTSIFLITIIGNGLVLFALYARRCYQRRKFTRMObiroiItr_B     MSYDLSSSSLPLSLSLPSSPSSSSSPSSLEVSDDLRDEYLARWEIAVLTSIFLITIIGNGLVLFALYARRCYQRRKFTRMObiroiItr_C     MSYDLSSSSLPLSLSLPSSPSSSSSPSSLEVSDDLRDEYLARWEIAVLTSIFLITIIGNGLVLFALYARRCYQRRKFTRMObiroiItr_D     MSYDLSSSSLPLSLSLPSSPSSSSSPSSLEVSDDLRDEYLARWEIAVLTSIFLITIIGNGLVLFALYARRCYQRRKFTRMObiroiItr_E     MSYDLSSSSLPLSLSLPSSPSSSSSPSSLEVSDDLRDEYLARWEIAVLTSIFLITIIGNGLVLFALYARRCYQRRKFTRMObiroiItr_F     MSYDLSSSSLPLSLSLPSSPSSSSSPSSLEVSDDLRDEYLARWEIAVLTSIFLITIIGNGLVLFALYARRCYQRRKFTRMObiroiItr_G     MSYDLSSSSLPLSLSLPSSPSSSSSPSSLEVSDDLRDEYLARWEIAVLTSIFLITIIGNGLVLFALYARRCYQRRKFTRMObiroiItr_H     MSYDLSSSSLPLSLSLPSSPSSSSSPSSLEVSDDLRDEYLARWEIAVLTSIFLITIIGNGLVLFALYARRCYQRRKFTRMObiroiItr_I     MSYDLSSSSLPLSLSLPSSPSSSSSPSSLEVSDDLRDEYLARWEIAVLTSIFLITIIGNGLVLFALYARRCYQRRKFTRMObiroiItr_J     MSYDLSSSSLPLSLSLPSSPSSSSSPSSLEVSDDLRDEYLARWEIAVLTSIFLITIIGNGLVLFALYARRCYQRRKFTRM                **** .:* *       *****   .**:*..** ************** ***:*:*** *:******** *********LnigerItr       YFFILHLSIADLLTGLLDVLPQLAWDITFRFQGGAVLCKLVKFGQPFGVYLSSYVLTVTAMDRYYAICHPFLYCSITSRRObiroiItr_A     YFFILHLSIADLLTGLLDVLPQLAWDITFRFQGGAVLCKLIKFGQPFGLYLSSYILTVTAMDRYYAICHPFSYCAVTSRRObiroiItr_B     YFFILHLSIADLLTGLLDVLPQLAWDITFRFQGGAVLCKLIKFGQPFGLYLSSYILTVTAMDRYYAICHPFSYCAVTSRRObiroiItr_C     YFFILHLSIADLLTGLLDVLPQLAWDITFRSS------------------------------------------------ObiroiItr_D     YFFILHLSIADLLTGLLDVLPQLAWDITFRSCFH----------------------------------------------ObiroiItr_E     YFFILHLSIADLLTGLLDVLPQLAWDITFRVARC----------------------------------------------ObiroiItr_F     YFFILHLSIADLLTGLLDVLPQLAWDITFRFQGGAVLCKLIKFGQPFGLYLSSYILTVTAMDRYYAICHPFSYCAVTSRRObiroiItr_G     YFFILHLSIADLLTGLLDVLPQLAWDITFRVARC----------------------------------------------ObiroiItr_H     YFFILHLSIADLLTGLLDVLPQLAWDITFRFQGGAVLCKLIKFGQPFGLYLSSYILTVTAMDRSS---------------ObiroiItr_I     YFFILHLSIADLLTGLLDVLPQLAWDITFRFQGGAVLCKLIKFGQPFGLYLSSYILTVTAMDRYYAICHPFSYCAVTSRRObiroiItr_J     YFFILHLSIADLLTGLLDVLPQLAWDITFRFQGGAVLCKLIKFGQPFGLYLSSYILTVTAMDRYYAICHPFSYCAVTSRR                ******************************                                                  LnigerItr       SKMMVYGAWTLAAILCVPQVFIFSYQEISPGVWECWATFYLKYGERAYVTWYSIMQFLLPFIVLVYTYT--KIC--IAIWObiroiItr_A     SKMMVYGAWVLAAILCVPQVFVFSYKEISPGVWECWATFYLKYGERAYITWYSVTQFLLPFIVLVYTYT--QIC--RSIWObiroiItr_B     SKMMVYGAWVLAAILCVPQVFVFSYKEISPGVWECWATFYLKYGERAYITC--VTQFLLPFIVLVYTYT--QIC--RSIWObiroiItr_C     -------------------------------------------------------------------YS--RIK--RYRRObiroiItr_D     -------------------------------------------------------------------YS--QSA--K---ObiroiItr_E     -----------------------------------------------------------------------SAN------ObiroiItr_F     SKMMVYGAWVLAAILSS--------------------------------------------------YS--RIK--RYRRObiroiItr_G     -----------------------------------------------------------------------SAN------ObiroiItr_H     -------------------------------------------------------------------YS--RIK--RYRRObiroiItr_I     SKMMVYGAWVLAAILCVPQVR-----------------------------------------------------------ObiroiItr_J     SKMMVYGAWVLAAILCV--------------------------------------------------PQTADANPTRRRR                                                                                                LnigerItr       TSSKMSGVVDLKKNNKANFSQRNREPLISKAMMNTVRQTIIVITLYIATSFPFIGSMLWATWDPKAFTLPFFTGAAFTILObiroiItr_A     TSGKMSGVVDFKQGNKASFSLRNRDPFISKALINTIKQTIVVVSLYIITSIPFIGCELWATWDPKASTSPFFTGAAFTILObiroiItr_B     TSGKMSGVVDFKQGNKASFSLRNRDPFISKALINTIKQTIVVVSLYIITSIPFIGCELWATWDPKASTSPFFTGAAFTILObiroiItr_C     ASGNA--------------------------------------------------------------------GPPFT--ObiroiItr_D     --------------------------------------------------------------------------------ObiroiItr_E     --------------------------------------------------------------------------------ObiroiItr_F     ASGNA--------------------------------------------------------------------GPPFT--ObiroiItr_G     --------------------------------------------------------------------------------ObiroiItr_H     ASGNA--------------------------------------------------------------------GPPFT--ObiroiItr_I     --------------------------------------------------------------------------------ObiroiItr_J     ANL-----------------------------------------------------------------------------                                                                                                LnigerItr       SLLNSLTSCVNPWIYFAFNKELRGALTNFFYRKKD-SLNYDIDARQNVSDVASTTSSFISRISRLASSKIFGObiroiItr_A     SLLNSLTSCVNPWIYFAFNRELRAALTNFFCRKKDYSLTYDIDAHQNASDVPSTTSSFISRISRLASSKIFGObiroiItr_B     SLLNSLTSCVNPWIYFAFNRELRAALTNFFCRKKDYSLTYDIDAHQNASDVPSTTSSFISRISRLASSKIFGObiroiItr_C     ------------------------------------------------------------------------ObiroiItr_D     ------------------------------------------------------------------------ObiroiItr_E     ------------------------------------------------------------------------ObiroiItr_F     ------------------------------------------------------------------------ObiroiItr_G     ------------------------------------------------------------------------ObiroiItr_H     ------------------------------------------------------------------------ObiroiItr_I     ------------------------------------------------------------------------ObiroiItr_J     ------------------------------------------------------------------------                                                                                        
