# Supplementary material for: An oxytocin/vasopressin-related neuropeptide modulates social foraging behavior in the clonal raider ant
Source: PLoS Biol. 2021 Jun 30;19(6):e3001305. doi: 10.1371/journal.pbio.3001305 (PMC8244912; doi:10.1371/journal.pbio.3001305)
Supplement: S1 Text — (DOCX) [file pbio.3001305.s022.docx]

**S1 Text**

**Supplementary methods**

**Calcium imaging of truncated isoform**

Experiments were conducted following Garrison J.L., et al 2012 [8] with modifications. Human embryonic kidney 293T cells (HEK293T) were cultured in DMEM and supplemented with 10% FBS at 37 °C in a cell incubator containing 5% CO^2^. Cells were grown in 35 mm MatTek glass bottom dishes to a level of 60 – 70% confluency. Cells were transfected with a 1:1 ratio of PCDNA3.3 containing ~ 1μg of *itcR* cDNA (full-length isoform A or truncated isoform H) (See S1 Fig for isoforms) and pcDNA3-alpha 16 (encoding promiscuous G protein 16) using Lipofectamine 2000. Cells were incubated for 24 h at 37 °C and then transferred to 28 °C for another 16-24 h. For control experiments, empty vector was also transfected. On the day of the experiment, the cells were loaded with 2.5 mM fura-2/AM (Invitrogen) for 25 min at 37 °C. Inotocin peptide diluted in Ringer’s solution was applied to the cells for 15 s with a peristaltic pump, and fluorescence at 510 nm was monitored (excitation at 340/380 nm) using a MetaFluor calcium imaging system with a Nikon Eclipse TE2000U microscope.

**Human oxytocin and vasopressin peptides used for Calcium experiments**

Oxytocin 66-0-52 (CYIQNCPLG-NH₂) and Arg-Vasopressin 66-0-03 (CYFQNCPRG-NH₂) (American peptide Company INC) peptides were used to perform calcium imaging experiments in cells transfected with the inotocin receptor.

**Differential gene expression analysis**

Gene expression data were analyzed from the control samples originally used in [52, 73]. Control samples in each case consist of age-matched workers in the reproductive phase in the presence of pupae, or age-matched workers in the brood care phase with larvae. Gene expression values were determined using the method described in [68]. Wald tests were performed on each group to calculate significance. Methods, scripts and data to produce these results have been published at https://github.com/oxpeter/ooceraea_inotocin_expression (DOI: 10.5281/zenodo.4562942).

Reference numbers given within the text of this document refer to the references list of the main manuscript. Supplementary References given below are referenced in S4 Fig.

**Supplementary References**

1. Jasper JR, Harrell CM, O'Brien JA, Pettibone DJ. Characterization of the human oxytocin receptor stably expressed in 293 human embryonic kidney cells. Life Sci. 1995;57(24):2253-2261.

2. Burger K, Fahrenholz F, Gimpl G. Non-genomic effects of progesterone on the signaling function of G protein-coupled receptors. FEBS Lett. 1999;464(1-2):25-29.

3. Ma WJ, Hashii M, Munesue T, Hayashi K, Yagi K, Yamagishi M, et al. Non-synonymous single-nucleotide variations of the human oxytocin receptor gene and autism spectrum disorders: a case-control study in a Japanese population and functional analysis. Mol Autism. 2013;4(1):22.

4. Zaelzer C, Gizowski C, Salmon CK, Murai KK, Bourque CW. Detection of activity-dependent vasopressin release from neuronal dendrites and axon terminals using sniffer cells. J Neurophysiol. 2018;120(3):1386-1396.

5. Adeyemo AA, Zaghloul NA, Chen G, Doumatey AP, Leitch CC, Hostelley TL, et al. ZRANB3 is an African-specific type 2 diabetes locus associated with beta-cell mass and insulin response. Nature communications. 2019;10(1):3195.
