# Supplementary material for: An oxytocin/vasopressin-related neuropeptide modulates social foraging behavior in the clonal raider ant
Source: PLoS Biol. 2021 Jun 30;19(6):e3001305. doi: 10.1371/journal.pbio.3001305 (PMC8244912; doi:10.1371/journal.pbio.3001305)
Supplement: Attachment — Submitted filename: Response to Reviewers_Inotocin_MS.pdf [file pbio.3001305.s023.pdf]

Response to Reviewers

We very much appreciate the comments of the three reviewers, which helped improve the manuscript substantially. Our responses to the specific issues raised by the reviewers are detailed below.

Sincerely,

Ingrid Fetter-Pruneda on behalf of all authors

REVIEWERS' COMMENTS:

Reviewer #1:

Summary

The author investigate regulation of social behavior by inotocin in the model ant system, O. biroi. First, they clone a full length isoform of the receptor and shows that it responds to the synthetic peptide when expressed in 293 cells. Then they perform IF with a custom antibody and FISH to identify two neurons that produce inotocin in the ant brain and their projections. Next, they show that levels of inotocin peptide (by IF) are higher in foragers and older ants. Finally, they use a pharmacological approach to show increased "foraging" in old ants in presence of larvae and young ants in presence of pupae.

Overall critique

The idea of studying inotocin in ants is interesting because of the important social roles of its orthologs in various animals and because, as the authors point out, Drosophila lacks this pathway. However, the current study adds little to our knowledge about this system. Figures 1-3 show results similar to those previously reported in other ant species and Fig. 4 show a confusing set of results using an assay that does not measure foraging behavior. Overall the study is preliminary and lacks novelty; therefore, I do not recommend its publication.

We thank the reviewer for providing feedback. The comments were very helpful in improving our manuscript.
We believe that our study is novel for four reasons. 1) We report the first inotocin gain of function experiments and quantifications at the peptide level in ants. Previous studies did not show differences at the peptide level between ants that perform different tasks and they did not study the behavioral effects of increasing peptide levels. 2) We show that inotocin modulates social behavior in clonal raider ants. Previous studies demonstrated a role for inotocin in locomotion and in cuticular hydrocarbon synthesis, but found no association with social behavior. Rather than simply affecting locomotion, we show that inotocin modulates behavior only in specific social contexts and in ants of a specific age group. Since the role of oxytocin in regulating social behavior seems to be evolutionarily conserved, showing that it is also involved in regulating social behavior in ants, which display complex social behavior, is very relevant. 3) We show that there are splicing variants of the receptor, which has not been previously reported, and we now report additional data showing that at least one of the variants does not elicit calcium responses. 4) We have now added *in situ* hybridization data that shows that the receptor is being expressed in the brain, including the subesophageal zone and the mushroom bodies, as well as in peripheral tissues and organs such as the ventral nerve cord, ovaries and Malpighian tubules,

consistent with a possible conserved role in regulating both behavior and physiology. We have also addressed the criticism that our assay does not measure foraging behavior (see our response to this reviewer's comment 5.2).

Major points

1. The abstract claims that the experimental evidence in this study suggest that "inotocin plays an important role in mediating age-polyethism". This is a big overstatement. The experiments show a correlation between age, foraging, and inotocin expression and no causal link with age polyethism.

We agree with the reviewer that our experiments do not show a causal link between inotocin expression and age polyethism. However, we show that pharmacologically increasing inotocin in older ants induces "foraging" in the presence of larvae, but not pupae. This is an important result since it shows that inotocin increases the behavioral response to social cues. We understand how our original phrasing was misleading and have rephrased it as:
"inotocin signaling plays an important role in modulating behaviors that correlate with age, such as social foraging ,"… on lines 44-45

2. Fig. 1C: missing controls, what about another neuropeptide receptor or at least the empty control mentioned in the methods? Also why not use the control shuffled peptide also mentioned in the methods?

We agree with the reviewer that controls were missing, and we have now included new supplementary figures that include the empty vector control, the control shuffled peptide, and cells stimulated with ATP as a test of cell health (Figure S2). These controls were run as part of the original experiments, but were not included in the original submission. We did not test another neuropeptide receptor, but we show that there is no response to inotocin when cells are transfected with an empty vector or with a truncated version of the receptor (Figure S5). We also added a supplementary figure showing that the inotocin receptor elicits calcium transients when stimulated with human oxytocin and vasopressin peptides (Figure S4).

3. Fig. 1-3: the first three figures recapitulate the data shown in Liutkeviciute & Gruber FASEB 2018 Fig. 1, 3, and 4. Although it is of some value to show that those conclusions hold in this different ant system, the novelty of these data is quite limited. I acknowledge that the authors detect protein levels rather than mRNA but that seems a small detail in this context.

We acknowledge that in previous studies in ants, differences in mRNA expression levels were reported. However, we believe that peptide levels constitute an important additional step in the context of the mechanisms regulating behavior and physiology. The previous three studies (Cherasse and Aron 2017, Liutkeviciute et al. 2018, Koto et al. 2019) focused on mRNA levels only and did not identify splice variants of the receptor, so the functional relevance of the differences they observe in the levels of gene expression remain to be fully addressed in the context of both increased peptide levels and functional receptor proteins. Moreover, Liutkeviciute et al. (2018) and Koto et al. (2019) provide only loss of function evidence. In contrast, in this manuscript we show the functional relevance of increased peptide levels, and we now also emphasize the importance and novelty of having discovered alternative splice variants of the receptor as an important avenue of future research. Furthermore, gain of function experiments in mammals for the oxytocin/vasopressin systems show that differences in peptide levels can have very strong effects on behavior, whereas loss of function mutants have subtle behavioral

defects (Neumann et al. 2010). Therefore, studies of mRNA alone do not show the full picture of what is happening at the physiological level.

4. Fig. 3C: I applaud the authors for showing this piece of evidence which does not fit well with the overall idea of inotocin regulating foraging but it does raise some important questions about the conclusions and the proposed model. What if they were to treat with inotocin the intercaste?

Intercastes seem to have different response thresholds to signals coming from the brood. They don't respond to the brood signals as much as the regular workers (Teseo et al. 2013, Chandra et al. 2018) and therefore we would expect them to be less responsive to the inotocin treatment than regular old workers. We thought this would overcomplicate the results, so we did not perform this experiment; however, this is an interesting avenue for future study.

5. Fig. 4 and S5: even though manipulation of the inotocin pathway in ants has been reported before (Liutkeviciute & Gruber 2018) this is the first gain-of-function experiment to my knowledge and therefore contains new information. Even so, this experiment is not complete and cannot be used to draw the conclusions that the authors wish to draw.
5.1. The treatment appears to be immersion of the ants in a inotocin-containing solution. Although creative, this seems highly unusual and should be openly mentioned in the description of the results rather than only in the material and methods. Further, it remains to be demonstrated that inotocin actually gets inside the ants, that it acts as an agonist in vivo at these concentrations, and that it acts in the brain. To the latter point, Liutkeviciute & Gruber noted that the inotocin receptor is broadly expressed and by delivering the peptide immersion it can be safely concluded that all receptors in the body will be activated.

We agree with the reviewer and have now added a sentence in the results section specifying that we performed whole body peptide immersion treatments (lines 275-276). *O. biroi* ants are very small, and we initially tried injecting them in the head instead of immersing them in the peptide. The damage produced by the injection directly affected the ants' behavior (ants started spinning in circles right after the treatment and eventually died). Therefore, we developed a less invasive method to deliver the pharmacological treatment. Topical treatment of insects and invertebrates in general with drugs or chemicals (including pesticides) is a common method of drug delivery (see for example: Barron et al. 2007 for a study in bees and Zheng et al. 2013 for a study in *C. elegans*). In the specific case of oxytocin, nasal sprays have been developed as a way of administering the peptide in humans to reach the brain (e.g. Quintana et al., 2015) and oxytocin topical treatments in rats have been shown to be effective (e.g. Rojas-Piloni et al. 2010). As the reviewer suggests however, the delivery method used in this paper could provide a means of reaching receptors anywhere in the ant, but so could peptide injected in the head. Moreover, our results show an effect in the ants' behavior only in the ants treated with inotocin. This result shows that the immersion protocol allows the peptide to reach its receptor and affect behavior. Together with our new data showing that the receptor is expressed in the brain (Figure 3), this suggests that inotocin from our immersion treatment does in fact reach those receptors.
Determining exactly how much peptide from the immersion treatment enters the ant and where exactly it acts is technically extremely challenging and beyond the scope of this manuscript.

5.2. The assay seems to measure general locomotion and not "foraging". In fact an effect of inotocin on locomotion was already reported by Liutkeviciute & Gruber. In my view, this cannot be considered a regulation of "social behavior".

We will address this concern in two parts. First, we explain why the reported behavioral effect is specifically social, and not simply an increase in locomotion (which is the most important part). Second, we will argue why it is appropriate and in line with other work in the field to talk about this effect as "foraging". However, we appreciate the reviewer's objection and have added additional clarifications to the text (detailed below).

i) If the inotocin treatment simply increased locomotor activity, we would expect there to be an effect in all ants. Our experimental design shows that increasing inotocin doesn't increase distance traveled in all ants, but is instead dependent on social context. Here, we show that old ants treated with inotocin increase the distance they travel ONLY in the presence of larvae, but not in the presence of pupae. Furthermore, the effect is contingent upon the age of the ant, parameters that we can precisely control in *O. biroi*. Therefore, in contrast to previous work, this experiment establishes a role for inotocin in modulating behavioral responses specifically to social cues. This corroborates our interpretation that we are measuring a social behavior and not locomotor activity per se.

ii) While the reviewer is correct that the behavioral metrics used here, namely root-mean-square deviation (r.m.s.d.) and average distance traveled, directly quantify the movement of ants in space, previous work has shown that, in this context, these metrics are biologically meaningful and reflect the propensity to perform tasks away from the nest (for example, foraging) rather than at the nest (for example, nursing). In the behavioral setup used here, r.m.s.d. increases when nutritional demand is experimentally elevated by increasing the larvae-to-workers ratio (Ulrich et al. 2018) showing that r.m.s.d. is an appropriate proxy for actual foraging behavior. The social regulation of adult foraging by the brood has been further demonstrated in two other publications (Ravary & Jaisson, 2006, Ulrich et al. 2016). We understand that not all activities performed outside the nest are foraging activities, and we have therefore clarified our definition of foraging behavior to avoid this confusion as follows:
"Given that tasks in insect societies are spatially organized (foraging, exploring and waste disposal occur away from the nest, whereas nursing occurs inside the nest), activity-related measures such as spatial location and distance travelled correlate well with foraging and are often used as proxies for foraging activity (e.g. Mersch et al. 2013, Ulrich et al. 2018, Koto et al. 2019)" lines 294-299.

5.3. The results are hard to interpret. Inotocin only has an effect on old ants with larvae and young ants with pupae? Even the authors seem unsure of what this means as "O biroi does not normally forage in the presence o pupae". If inotocin is normally already high in old workers but low in young workers, wouldn't one expect a stronger effect in the latter? Are there differences in the receptor?

We would not necessarily expect stronger effects in younger workers because they normally have lower inotocin levels. It looks like higher levels of inotocin signaling aren't as important in young ants as in older ants. As the reviewer suggests, it could be that the levels and sites of expression of the receptor and the isoforms expressed might be involved in the different responses in young and old ants. Our data show that distance traveled only increases in old ants in colonies with larvae and not in colonies with pupae, showing that inotocin modulates the response to social cues. We also show that young ants in colonies with pupae respond to the inotocin treatment. We currently do not know the reason for this response, considering that ants in colonies with pupae do not normally forage. Therefore, as discussed in the paper, this result would require follow up experiments to analyze the behavioral response of young ants to the peptide.

5.4. Finally, the different "social contexts" are only introduced in this very last experiment. Why not also in the analysis of inotocin expression in Fig. 3? And what about receptor expression? Is it possible that different social contexts regulate receptor expression rather than inotocin itself?

In response to this comment, we reanalyzed published whole brain expression data in *Ooceraea biroi* (Libbrecht et al. 2016 and Libbrecht et al. 2018) and did not find evidence of changes in gene expression for the peptide or receptor genes during the reproductive- vs. the brood care phase, during which ants are in different social contexts (i.e. with pupae vs. with larvae). We have now added this data as figure S11. This is consistent with the idea that gene expression is regulated differentially in specific behavioral castes (e.g. old vs. young; age-matched nurses vs. foragers), but no necessarily as a function of the immediate social context (which again is consistent with the idea of behavioral response thresholds, which should be stable over short time periods). We therefore believe that behavioral differences are mostly due to differences in inotocin levels between behavioral “subcastes” (especially different age cohorts), and that the inotocin receptor might have related effects, which is something we plan to study in more detail in the future. However, it is possible that the social context does in fact lead to more subtle changes in peptide or receptor expression that we were unable to detect in our current experiments. This is also something worth exploring more in the future.

Minor point
- why are the legends interspersed with the text even if the figures are at the end?
This is the format that the journal asked for.

- Fig. 1B: the legend states that the transmembrane domains are "required for receptor function". This needs experimental support or a citation. If the authors are correct it seems that 8/10 of the isoforms would be nonfunctional.
The reviewer is correct that we expect 8/10 isoforms to be “non-functional” based on data from studies on the human oxytocin receptor (Gimpl and Fahrenholz 2001). According to Gimpl and Fahrenholz (2001), there are important sites for the binding of oxytocin, peptide docking sites and residues required for Gq protein coupling beyond transmembrane domain 3 (TM3). Moreover, a recent crystal structure of the OxtR was published and an important site for extrahelical cholesterol binding was found between helices IV and V of the human oxytocin receptor, which seems to be crucial for OTR function (Waltenspuhl et al. 2020).
Eight out of the ten splice variants that we found are missing the predicted binding site for the cyclic portion of the inotocin peptide, peptide docking sites, as well as the residues required for coupling to Gq protein; therefore, we expect them to be “non-functional”. We have added the two citations mentioned to support this claim (lines 126-127). Nevertheless, the functional relevance of the truncated versions remains to be thoroughly studied, since truncated versions could still play a role in the regulation of inotocin signaling. Splice variants might function to down-regulate the activity of the full-length receptor as a dominant negative: competing for ligand or dimerizing with and retaining the full-length receptor in the endoplasmic reticulum (e.g. Wise 2012). We have added new data showing that a splice variant of the receptor that only produces the first three transmembrane domains does not show calcium responses when stimulated with inotocin (Figure S5), and we have expanded on our discussion of the possible functional relevance of these “non-functional” splice variants (lines 126-133).

- Fig. 4B: it is unclear if these example tracks are from colonies with larvae, pupae etc.
These tracks are from colonies with larvae, which was indicated in the figure legend. For clarity, we have now added a label to the side of the figure.

Reviewer #2:

This thorough study of inotocin in the Ooceraea clonal raider ant demonstrates that inotocin signaling is likely to be a conserved feature of ant biology with the potential to regulate responsiveness to social cues and division of labor. The methodology and experimental design are impressive and appear robust, including the commendable use of assays of peptide levels as well as treatments with the inotocin peptide in combination with the tracking of individual ant movements in different social contexts. The results of the study build on and recapitulate findings from two recent studies conducted in ants from a different subfamily. Among the key findings here are that inotocin stimulates foraging behavior (more movement throughout an arena) and that this effect interacts with age and social stimulus (particularly the presence of larvae that need feeding). Among the open questions are where the inotocin receptor localizes in Ooceraea and whether the localization is consistent
with its function in the brain and/or with its function in desiccation resistance observed in Camponotus ants, which would provide very interesting insight into questions of evolutionary lability and pleiotropy of inotocin signaling. Such insight would undoubtedly raise the impact of the current study, which I think would be the main question in whether it rises to the level of PLOS Biology, but the findings are impressive in the current form. In reading the manuscript and the other two studies from the formicine ants I learned some interesting things and would consider this time well spent for anyone interested in the proximate underpinnings of the social lives of ants. I would simply suggest that the title should be revised - if "in ants" is changed to "in an ant" or the title is otherwise softened it may more appropriately characterize the incomplete nature of the picture of the generalities of the roles of inotocin signaling in ants at present.

We thank the reviewer for providing these insights. We have now included *in situ* hybridization data on the localization of the inotocin receptor (*itcR*) (the new Figure 3). We find staining in the brain, dorsal nerve cord, Malpighian tubules and ovaries. These patterns are consistent with inotocin's possible role in regulating both behavior and physiology. We did not stain fat bodies and therefore cannot comment on the presence/absence of the receptor in those cells, but a thorough characterization of the receptor will be the scope of future research.

As suggested by the reviewer we have changed the title to:
"An oxytocin/vasopressin-related neuropeptide modulates social foraging behavior in the clonal raider ant"

Reviewer #3:

The manuscript entitled "Oxytocin/vasopressin-related peptide modulates foraging in ants" by Fetter-Pruneda et al. describes a role for the neuropeptide inotocin in regulating division of labor in colonies of the clonal raider ant. While multiple different papers have demonstrated associations between several signaling pathways and worker's division labor in multiple social insect species, this well-written manuscript is one of very few that actually demonstrate a possible causal link between a conserved peptidergic pathway and the neural regulation of a derived social behavioral trait. Below are more specific comments that I hope the authors will find useful:

1. It is possible that regulation of the inotocin receptor plays as important role as the ligand in regulating DOL in this species. Since the gene encoding it has been identified, and the authors can generate beautiful ISH data, it could be very informative to examine is expression as well. At minimum,

the authors should discuss in more depth what is known about the regulation of peptidergic systems, and should probably include a discussion of how the regulation of peptide secretion and receptor signaling could play a role in regulating behavior independent of quantitative changes in ligand expression.

We thank the reviewer for the very helpful comments. We have included a new figure that shows the sites of expression of the receptor through *in situ* hybridization (Figure 3). We agree that the receptor may play as important a role as the ligand in regulating division of labor in *O. biroi* ants. Not only the levels of expression but the site and splicing versions of the receptor may regulate inotocin's actions. We have added more information to highlight this point (lines 126-133). Moreover, in mammals, the site of expression of the receptor is very relevant for the expression of behavior (e.g. polygamous and monogamous voles (Insel and Shapiro 1992) and expression in the auditory cortex of mice or rats responding to pup calls (Marlin et al. 2015)). We have added a sentence in the discussion to further emphasize this important point (Lines 390-395).

2. Demonstrating that the candidate inotocin receptor show response to the ligand in physiologically-relevant concentrations is an important aspect of this study. However, since the authors have used human HEK293 cells to express an insect GPCR, it is somewhat surprising that they were able to observe a response without the co-transfection of a promiscuous Gqα subunit. Adding cAMP activation data could be one way to further convince readers that the measured intracellular signals are not somehow artifactual. Adding images showing HEK cells at baseline and post-activation would further increase confidence in the approach.

We agree with the reviewer's assessment and have now included new figures that show additional baseline controls (scrambled peptide and vehicle) as well as the receptor co-transfected with various G proteins (Figures S2 and S3). The oxytocin receptor has been reported to couple to Gqα11 type protein subunits but also to Gi and Go protein complexes (McKay and Counts 2020). Therefore, when we characterized the ItcR receptor activity *in vitro*, we co-transfected the receptor with three different G proteins: a promiscuous Gqα (Gqα16) and two chimeric proteins (Gqαi and Gqαo (Coward et al. 1999)) and with an empty vector. The ItcR transfected with the empty vector alone elicited an inotocin response in a dose dependent manner. Therefore, we hypothesize that the receptor is signaling through an endogenous G protein in the HEK293 cells. These results did not allow us to gain more insight into which types of G proteins are required for inotocin signaling. We believe that by using different cell lines, G protein null mutant cell lines, or pharmacological strategies we will be able to shed light on this important aspect of inotocin signaling. However, this is beyond the scope of the current manuscript.

3. Is it possible that inotocin injections induce general hyperactivity, which might be interpreted as increased foraging? It seems it would be possible to extract the rates of individual activity from the tracking data the authors already have.

We appreciate the reviewer's concern, which was shared with reviewer 1.
In our response to reviewer 1, comment 5.2, we explained why the observed behavioral effect is specifically social, and not simply an increase in locomotion or in this case general hyperactivity and we also explain why we believe it is appropriate to call it foraging behavior.
We show that only the ants treated with inotocin and of a specific age group and in a specific social context showed increased foraging. If the treatment induced general hyperactivity, it should do so irrespective of social context and age.

4. Adding high-res images of stained brains from young nurses and old foragers will allow readers to appreciate the observed differences qualitatively.

We thank the reviewer for this suggestion. We have now added representative images of the cell bodies that allow the observation of the differences in inotocin peptide qualitatively.

5. It's not clear if the authors tracked injected animals only or all colony members. If all then they should also show the tracking data for untreated animals. For example, it would be very interesting to know if increased foraging in older injected animals increased the activity in the non-focal older ants.

We tracked all animals in the colony and have made this clearer in the methods section (Lines 707-708). We didn't detect increased activity in untreated ants, beyond the initial transient relative increase while experimental ants were still recovering from the immersion treatment. We have now added the data of the untreated ants to Figure S10.
