# Supplementary material for: An oxytocin/vasopressin-related neuropeptide modulates social foraging behavior in the clonal raider ant
Source: PLoS Biol. 2021 Jun 30;19(6):e3001305. doi: 10.1371/journal.pbio.3001305 (PMC8244912; doi:10.1371/journal.pbio.3001305)
Supplement: Attachment — Submitted filename: Fetter_Pruneda_Response_Reviewers.pdf [file pbio.3001305.s024.pdf]

Instituto de Investigaciones Biomédicas

Código Postal 04510
Ciudad Universitaria, Ciudad de México

Response to Reviewers

Reviewer 1:

We would like to thank this reviewer for their additional comments that further improved the quality of our manuscript.

- Lines 47-8: I don't think the authors demonstrated that "Inotocin signaling [...] contributes to behavioral individuality and division of labor in ant societies." I suggest to tone this last sentence down.

We have edited this last sentence of the abstract to: "Inotocin signaling thereby likely contributes to behavioral individuality and division of labor in ant societies."

-The authors added important controls for Fig 1C in the supplement, but they also present new data in Fig. S4 that lack proper controls. Are the authors claiming that the O. biroi itcR binds and respond to human vasopressin and oxytocin? If so an empty vector control must be shown to exclude that the 293 cells used respond naturally to these neuropeptides.

We agree with the reviewer that these are important controls. However, published data shows that untransfected and mock transfected (empty vector) HEK293 cells do not show calcium responses when stimulated with oxytocin (Burger, Fahrenholz et al. 1999, Ma, Hashii et al. 2013) or vasopressin (Burger, Fahrenholz et al. 1999). In addition, their receptors are not detected with antibodies in untransfected HEK293 cells (Ma, Hashii et al. 2013, Zaelzer, Gizowski et al. 2018), and radiolabeled oxytocin and vasopressin do not bind to untransfected HEK293 cell membranes (Jasper, Harrell et al. 1995). Therefore, HEK293 cells do not seem to express the oxytocin or vasopressin receptors endogenously, and we did not think that repeating these controls was necessary. Our results do show that the *O. biroi* itcR binds and responds to the human vasopressin and oxytocin peptides. This discovery is not without precedent; the beetle inotocin receptor responds to human oxytocin in CHO cells (Stafflinger, Hansen et al. 2008), and CHO cells transfected with itcR from *Lasius* ants also respond to human oxytocin (Koto, Motoyama et al. 2019).

In response to the reviewer, we have added the following sentence in the legend of figure S4: "Previous experiments have shown that HEK293 cells do not endogenously express the

oxytocin or vasopressin receptors (Jasper, Harrell et al. 1995, Burger, Fahrenholz et al. 1999, Ma, Hashii et al. 2013, Zaelzer, Gizowski et al. 2018)”.

-Fig.S5: possibly due to my own lack of familiarity with this technique I cant' interpret these microscopy images. Why not show a quantification as in Fig. 1C, Fig. S3, 4?
The transfected cells were loaded with a calcium sensitive dye that when excited at 340/380 nm emits a signal at 510 nm (hence the red color when there is a calcium response). The inotocin peptide was then applied to the cells with a peristaltic pump and the fluorescence was monitored using an epifluorescence microscope equipped with a calcium imaging system. The figure shows the cells before and after stimulus with the inotocin peptide or control.

The experiment shown in Fig. S5 was performed using a different setup from the one used in Figs. 1C, S3 and S4. We used the setup for Fig. S5 for the initial characterization of the receptor, and for the scope of this paper the quantitative characterization of the receptor isoform was not necessary and therefore we did not repeat this condition in our more high-throughput/quantitative experiments. Characterizing all receptor isoforms in a quantitative way could become the scope of future research.

Reviewer 3:
We would again like to thank the reviewer for their very helpful comments in the review of the initial submission, and we extend additional thanks for their positive assessment of the revised manuscript.
